# Postoperative Evaluation of Pain and Disability in Patients Undergoing Spinal Discectomy

**DOI:** 10.7759/cureus.49963

**Published:** 2023-12-05

**Authors:** Kumar Abhinav, Dikpal Jadhav, Arun Kumar Agrawal, Rama Agrawal, Ankita Agrawal

**Affiliations:** 1 Department of Neurosurgery, Lilavati Hospital and Research Centre, Mumbai, IND; 2 Department of Neurosurgery, King Edward Memorial (KEM) Hospital, Mumbai, IND; 3 Department of Neurosurgery, Patna Medical College and Hospital, Patna, IND; 4 Department of Physiology, Patna Medical College and Hospital, Patna, IND; 5 Department of Conservative Dentistry and Endodontics, Buddha Institute of Dental Sciences and Hospital, Patna, IND

**Keywords:** spinal discectomy, neurosurgery, disability, pain, postoperative patterns

## Abstract

Background: A spinal discectomy surgery (SDS) is a common surgical procedure performed to treat lumbosacral radiculopathy.

Aim: To evaluate postoperative patterns of pain and disability in patients undergoing spinal discectomy.

Methods and materials: This investigation was a retrospective longitudinal review of prospective information gathered from 543 enrolled patients for lumbar radiculoplasty. The study participants were divided into two categories: Category 1 (SDS) comprising patients of lumbar radiculoplasty managed with SDS (n=270) and Category 2 (non-SDS) comprising patients of lumbar radiculoplasty managed with therapy other than SDS (n=273). It included study participants taking medication for pain control including opioids and non-opioids and physiotherapy for strengthening lower back muscles. At baseline, three months, 12 months, and 24 months after surgery, patient-reported information was gathered. Leg pain magnitude, back pain magnitude, and pain-related impairment were the key outcome metrics of interest.

Results: The mean postoperative visual analog scale (VAS) score for leg pain at three-month follow-up was 4.3±1.2 in study participants in SDS and 8.1±1.3 in the non-SDS category. The VAS score was lower in the SDS category showing greater reduction in postoperative pain with statistically meaningful results (p<0.001). The mean postoperative VAS score at 12-month follow-up was 2.8±1.1 in study participants in SDS and 7.9±1.5 in the non-SDS category. The VAS score was lower in the SDS category showing greater reduction in postoperative pain with statistically meaningful results (p<0.001). The mean postoperative VAS score at 24-month follow-up was 1.7±1.2 in study participants in SDS and 7.1±1.1 in the non-SDS category. The VAS score was lower in the SDS category showing greater reduction in postoperative pain with statistically meaningful results (p<0.001).

Conclusion: It was observed that after discectomy, patients suffering from lumbar radiculopathy have significant pain and disability recovery. According to these results, only a small percentage of individuals exhibit negative results at the level of impairment.

## Introduction

Lumbosacral radiculopathy (LR), often known as sciatica, is a spinal dysfunction symptom that is characterized by back/buttock pain spreading into the lower extremities and may also include muscular weakness along with sensory disorders [1-3]. The most common cause of LR is disease-related entrapment of the spinal nerves in the discs that connect the vertebrae or other structures [4-6]. Men and women aged 45-64 years had an estimated frequency of LR ranging from 1.6% to 13.4% [7,8].

A popular surgical treatment used for the management of LR brought on by disc pathology is a spinal discectomy surgery (SDS) [9]. An SDS is now preferred over non-surgical procedures because of pain alleviation and improved functioning in the initial phase, compared to at the halfway or for a long-term follow-up [10].

Although there is some variation in outcome, SDS is regarded as being a highly beneficial therapy for a large number of patients, carried out utilizing either a surgical procedure that is minimally invasive or an invasive open technique [6-11]. According to research, approximately 30-70% of patients say that pain persists after surgery, indicating that plenty of people do not have a positive postoperative result trajectory [12,13]. Multiple impairments and pain trends might be present within a larger patient group having SDS, according to studies conducted in other surgical procedures of the spine including decompression for spinal stenosis [14-19].

The most prevalent type of pain among adults is low back pain (LBP), which affects eight out of 10 people at some point in their lifetime. According to recent studies, LBP is becoming more common and costs associated with it are rising [1,2]. A kind of low back problem called lumbar disc herniation occasionally calls for surgery. However, because of constant discomfort, disability, and a reduced standard of life which happen in some people after the procedure, the clinical effects of disc surgery have been characterized as being less than ideal [3,4]. As a result, some people have said that unsuccessful disc surgery is a significant healthcare issue. Therefore, it has been decided that improving clinical results after lumbar disc surgery should be a top goal for future study. While the technical aspects of the surgical methods have garnered attention, less emphasis has been paid to potentially significant muscular deficits and clinical issues in the postoperative treatment of this population.

The specific goal of the present investigation is to evaluate postoperative pain and impairment patterns in individuals who received SDS for the management of LR.

## Materials and methods

Study design and participants

This investigation was a retrospective longitudinal review of prospective information gathered from 543 enrolled patients with a diagnosis of lumbar radiculoplasty at Lilavati Hospital and Research Centre, Mumbai, India, from January to October 2018. The study participants were divided into two categories: Category 1 (SDS) comprising patients of lumbar radiculoplasty managed with SDS (n=270) and Category 2 (non-SDS) comprising patients of lumbar radiculoplasty managed with therapy other than SDS (n=273). It included study participants taking medication for pain control including opioids and non-opioids and physiotherapy for strengthening lower back muscles.

The following were the criteria for inclusion: study participants at least 18 years old, the diagnosis of lumbar radiculopathy, and study participants who underwent SDS with open or minimally invasive surgical methods for degenerative disease and patients of lumbar radiculoplasty managed with therapy other than SDS that included those taking any medication for pain control including opioids and non-opioids and physiotherapy for strengthening lower back muscles. In contrast, exclusion criteria include individuals exhibiting pathology more than degeneration (such as fracture, cancer, or illness).

Measures of interest

Preliminary clinical as well as demographic information was gathered from the study participants before surgery and the beginning of therapy. Age in years, gender, habit of smoking and BMI prior to surgery on the spine, duration of illness, various comorbidities like diabetes, hypertension, renal osteodystrophy, etc., and number of vertebrae operated on were among these characteristics. Leg pain magnitude, back pain magnitude, and pain-related impairment were the key outcome metrics of interest.

Leg pain and back pain were analyzed with the visual analog scale (VAS), while pain-related impairment was assessed using the modified Oswestry Disability Index (ODI) [20-21]. On a scale from 0 to 100, higher scores indicated greater disability. Total scores were divided into five categories: minimal disability=0-20, moderate disability=21-40, severe disability=41-60, crippled=61-80, and bedbound or exaggerating=81-100 [21]. At baseline, three months, 12 months, and 24 months after surgery, patient-reported information were gathered.

Statistical analysis

IBM SPSS Statistics for Windows, Version 28.0 (Released 2021; IBM Corp., Armonk, New York, United States) was used for all analyses. Categorical variables were represented with percentages, while continuum factors were represented as mean±standard deviation (SD). Two-way analysis of variance (ANOVA) and Pearson coefficient analysis were carried out. A p-value of <0.05 was considered statistically significant.

## Results

The demographic details of study participants are represented in Table 1.

**Table 1:** Demographic characteristics of lumbar radiculopathy patients SD: standard deviation; SDS: spinal discectomy surgery

Characteristic	Age (mean±SD) (years)	Male patients	Smoker	Mean BMI
SDS	60±17	58%	29%	27±10
Non-SDS	60±19	49%	30%	28±14
p-value	0.74	0.49	0.67	0.42

The mean age of study participants in the category of SDS was 60±17 years, while that of non-SDS study participants was 60±19 years. It was observed that males constituted 58% of the total study participants in the SDS category while 49% of study participants in the non-SDS category were males. Twenty-nine percent of study participants in the SDS category were smokers, while 30% of study participants were smokers in the non-SDS category. The mean BMI in SDS patients was 27±10, while the mean BMI was 28±14 in non-SDS participants. The demographic details were comparable in both SDS and non-SDS participants with no major variation statistically.

The mean VAS score for back pain preoperatively was 6.3±1.5 in SDS study participants, while the mean VAS score in non-SDS study participants was 6.3±1.43. The mean VAS score for leg pain preoperatively was 8.3±1.3 in SDS study participants, while the mean VAS score in non-SDS study participants was 8.4±1.2. The mean ODI score in SDS study participants preoperatively was 51±13, while the mean ODI score in non-SDS study participants was 51±13. The difference in findings was not meaningful statistically (Table 2).

**Table 2:** Pain ratings and disability ratings at baseline SDS: spinal discectomy surgery; ODI: Oswestry Disability Index; VAS: visual analog scale

Pain/disability score	Mean VAS back pain	Mean VAS leg pain	Mean ODI
SDS	6.3±1.5	8.3±1.3	51±13
Non-SDS	6.3±1.43	8.4±1.2	51±13
p-value	0.95	0.53	0.82

The mean postoperative VAS score for back pain at three-month follow-up was 5.1±1.7 in study participants in SDS, while it was 5.9±1.5 in the non-SDS category. The VAS score was lower in the SDS category showing greater reduction in postoperative pain with statistically meaningful results (p=0.04). The mean postoperative VAS score at 12-month follow-up was 4.4±1.7 in study participants in SDS, while it was 5.5±1.5 in the non-SDS category. The VAS score was lower in the SDS category showing greater reduction in postoperative pain with statistically meaningful results (p=0.02). The mean postoperative VAS score at 24-month follow-up was 3.8±1.7 in study participants in SDS, while it was 5.1±1.5 in the non-SDS category. The VAS score was lower in the SDS category showing greater reduction in postoperative pain with statistically meaningful results (p=0.01).

The mean postoperative VAS score for leg pain at three-month follow-up was 4.3±1.2 in study participants in SDS, while it was 8.1±1.3 in the non-SDS category. The VAS score was lower in the SDS category showing greater reduction in postoperative pain with statistically meaningful results (p<0.001). The mean postoperative VAS score at 12-month follow-up was 2.8±1.1 in study participants in SDS, while it was 7.9±1.5 in the non-SDS category. The VAS score was lower in the SDS category showing greater reduction in postoperative pain with statistically meaningful results (p<0.001). The mean postoperative VAS score at 24-month follow-up was 1.7±1.2 in study participants in SDS, while it was 7.1±1.1 in the non-SDS category. The VAS score was lower in the SDS category showing greater reduction in postoperative pain with statistically meaningful results (p<0.001).

The mean postoperative ODI score at three-month follow-up was 35±12 in study participants in SDS, while it was 49±13 in the non-SDS category. The ODI score was lower in the SDS category showing greater reduction in postoperative pain with statistically meaningful results (p<0.001). The mean postoperative ODI score at 12-month follow-up was 27±12 in study participants in SDS, while it was 41±14 in the non-SDS category. The ODI score was lower in the SDS category showing greater reduction in postoperative pain with statistically meaningful results (p<0.001). The mean postoperative ODI score at 24-month follow-up was 23±11 in study participants in SDS, while it was 34±14 in the non-SDS category. The ODI score was lower in the SDS category showing greater reduction in postoperative disability with statistically meaningful results (p<0.001). It was also observed that there was reduction in postoperative pain and postoperative disability in participants of both the SDS category and non-SDS category; however, the changes in the non-SDS category was nonsignificant statistically (Table 3 and Table 4).

**Table 3:** Postoperative pain and disability scores at different follow-up SDS: spinal discectomy surgery; ODI: Oswestry Disability Index; VAS: visual analog scale

Pain/disability score	Mean VAS back pain	Mean VAS leg pain	Mean ODI
Three months			
SDS	5.1±1.7	4.3±1.2	35±12
Non-SDS	5.9±1.5	8.1±1.3	49±13
p-value	0.04	<0.001	<0.001
12 months			
SDS	4.4±1.7	2.8±1.1	27±12
Non-SDS	5.5±1.5	7.9±1.5	41±14
p-value	0.02	<0.001	<0.001
24 months			
SDS	3.8±1.7	1.7±1.2	23±11
Non-SDS	5.1±1.5	7.1±1.1	34±14
p-value	0.01	<0.001	<0.001

**Table 4:** Postoperative pain and disability scores in both categories SDS: spinal discectomy surgery; ODI: Oswestry Disability Index; VAS: visual analog scale

Pain/disability score	Mean VAS back pain	Mean VAS leg pain	Mean ODI
SDS			
Three months	5.1±1.7	4.3±1.2	35±12
12 months	4.4±1.7	2.8±1.1	27±12
24 months	3.8±1.7	1.7±1.2	23±11
p-value	0.02	0.01	0.004
Non-SDS			
Three months	5.9±1.5	8.1±1.3	49±13
12 months	5.5±1.5	7.9±1.5	41±14
24 months	5.1±1.5	7.1±1.1	34±14
p-value	0.143	0.232	0.414

## Discussion

SDS is considered to be a highly helpful therapy for a significant number of patients, whether performed with a surgical process that is minimally invasive or extensive open technique, despite there being some difference in outcome [15-16]. According to the study, between 30% and 70% of patients report that their pain persisted after surgery, indicating that many patients did not experience good postoperative outcomes [17-18]. According to research done on other spinal surgical procedures, such as decompression for spinal stenosis [19], several disabilities and pain patterns may be present within a larger patient group with SDS. The condition LR, sometimes referred to as sciatica, is a spinal dysfunction symptom that is described by lower extremity pain radiating from the back and buttocks and may also include sensory and muscle problems. Entrapment of spinal nerves due to illness in vertebral discs or other structures is the most typical cause of LR [20-23]. An estimated frequency of LR in men and women aged 45-64 years ranged from 1.6% to 13.4% [24-25].

A lumbar discectomy is a common surgical procedure performed to treat LR brought on by disc disease. Because of the immediate pain relief and increased functioning compared to the intermediate- or long-term follow-up, lumbar discectomy is now favored over non-surgical therapies [20-26]. The current investigation's specific objective was to assess postoperative pain and disability patterns in those who got SDS.

In this study, the VAS score for back pain was lower in the SDS category showing greater reduction in postoperative pain with statistically meaningful results (p=0.01). According to the present research, the mean postoperative VAS score for back pain at three-month follow-up was 5.1±1.7 in study participants in SDS, while it was 5.9±1.5 in the non-SDS category. The VAS score was lower in the SDS category showing greater reduction in postoperative pain with statistically meaningful results (p=0.04). The mean postoperative VAS score at 12-month follow-up was 4.4±1.7 in study participants in SDS, while it was 5.5±1.5 in the non-SDS category. The VAS score was lower in the SDS category showing greater reduction in postoperative pain with statistically meaningful results (p=0.02). The mean postoperative VAS score at 24-month follow-up was 3.8±1.7 in study participants in SDS, while it was 5.1±1.5 in the non-SDS category. The VAS score was lower in the SDS category showing greater reduction in postoperative pain with statistically meaningful results (p=0.01).

According to a most recent meta-analysis and systematic review, patients' leg discomfort after SDS undergoes a substantial reduction that is subsequently preserved over time [27-28]. Investigations of people who had lumbar disc surgery produced comparable findings. A representative group of patients, three months after lumbar disc surgery, were reported to have structural and functional impairments of the lumbar multifidus by Mayer and colleagues [27,29]. The scientists noted diminished trunk extension strength, lower muscle density (signifying a larger proportion of intramuscular fat), and decreased paraspinal cross-sectional area. Similar to this, additional studies have documented lumbar multifidus muscle atrophy and damage after lumbar disc surgery, albeit these findings seem to depend on the surgical technique and length of intraoperative retraction. Additionally, these modifications might be connected to the emergence of failing back syndrome or post-discectomy syndrome.

Recommendations for activity limitation are another component of patient treatment after lumbar disc surgery. Activity limitations after lumbar disc surgery are extremely variable and may be brought on by physicians' worries about recurrent injuries, reherniation, or destabilization [12,13]. Some people are worried that overly cautious suggestions for returning to activities and a focus on the risk of reinjury will cause patients' recovery from disc surgery to be delayed. The implementation of postoperative constraints in this population does not appear to have an evidence-based reason, but there is some evidence that suggests that recovery may be improved if there are no activity restrictions after lumbar disc surgery [14,15].

In this study, the mean postoperative VAS score for leg pain at three-month follow-up was 4.3±1.2 in study participants in SDS, while it was 8.1±1.3 in the non-SDS category. The VAS score was lower in the SDS category showing greater reduction in postoperative pain with statistically meaningful results (p<0.001). The mean postoperative VAS score at 12-month follow-up was 2.8±1.1 in study participants in SDS, while it was 7.9±1.5 in the non-SDS category. The VAS score was lower in the SDS category showing greater reduction in postoperative pain with statistically meaningful results (p<0.001). The mean postoperative VAS score at 24-month follow-up was 1.7±1.2 in study participants in SDS, while it was 7.1±1.1 in the non-SDS category. The VAS score was lower in the SDS category showing greater reduction in postoperative pain with statistically meaningful results (p<0.001).

Despite the fact that there is evidence to support the benefits of rehabilitation after lumbar disc surgery, its application seems to be low, and there is little agreement among doctors and academics regarding the best practices [16,17]. As a result, it is difficult to determine the best strategy for rehabilitating this population. However, a review of pertinent randomized trials looking at the effectiveness of rehabilitation after lumbar disc herniation surgery finds two similar characteristics among successful trials [18,19]. First, lumbar stabilization exercises received priority attention. Second, compared to negative trials, positive trials appeared to begin rehabilitation sooner in the recovery period (about four versus seven weeks) [20,21].

In this study, the mean postoperative ODI score at three-month follow-up was 35±12 in study participants in SDS, while it was 49±13 in the non-SDS category. The ODI score was lower in the SDS category showing greater reduction in postoperative pain with statistically meaningful results (p<0.001). The mean postoperative ODI score at 12-month follow-up was 27±12 in study participants in SDS, while it was 41±14 in the non-SDS category. The ODI score was lower in the SDS category showing greater reduction in postoperative pain with statistically meaningful results (p<0.001). The mean postoperative ODI score at 24-month follow-up was 23±11 in study participants in SDS, while it was 34±14 in the non-SDS category. The ODI score was lower in the SDS category showing greater reduction in postoperative disability with statistically meaningful results (p<0.001). It was also observed that there was reduction in postoperative pain and postoperative disability in participants of both the SDS category and non-SDS category; however, the changes in the non-SDS category were nonsignificant statistically.

Even though results can vary, SDS, whether performed using a minimally invasive or invasive open technique, is seen to be a highly helpful therapy for a substantial number of patients in our study [13]. However, in a previous study, many patients do not have a great postoperative outcome, as evidenced by the research's finding in which 30-70% of patients report that pain continues after surgery. According to research done on other spinal surgical procedures, such as decompression for spinal stenosis, disability and pain may be evident within a larger patient group suffering SDS [14].

## Conclusions

It was observed that after discectomy, patients suffering from lumbar radiculopathy have significant pain and disability recovery. According to these results, only a small percentage of individuals exhibit negative results at the level of impairment.
